# Fatal Non‐Hepatic Hyperammonemia Post‐Glofitamab: *Ureaplasma* and Genetic Susceptibility: A Case Report

**DOI:** 10.1002/iid3.70443

**Published:** 2026-04-20

**Authors:** Yinshan Wu, Xiuliu Guo, Xiaoling Wang, Feng Guo

**Affiliations:** ^1^ Department of Critical Care Medicine, Sir Run Run Shaw Hospital, School of Medicine Zhejiang University Hangzhou Zhejiang China; ^2^ Nursing Department, Sir Run Run Shaw Hospital Zhejiang University School of Medicine Hangzhou China

**Keywords:** case report, hematologic malignancies, non‐hepatic hyperammonemia, SLC25A13 variant, *Ureaplasma urealyticum*

## Abstract

**Background:**

Although primarily reported in solid organ transplant recipients and patients undergoing chimeric antigen receptor T‐cell immunotherapy (CAR‐T), non‐hepatic hyperammonemia (NHHA) is a rare but lethal complication in the broader context of post‐ chemo‐immunotherapy hematologic malignancies. It often presents with unexplained encephalopathy that mimics primary central nervous system (CNS) progression, leading to diagnostic delays. With the expanding use of bispecific antibodies (e.g., glofitamab), the etiology of NHHA, particularly the complex interplay between opportunistic infections and potential metabolic susceptibility, remains poorly understood.

**Case Presentation:**

We report a fatal case of NHHA in a 58‐year‐old male with diffuse large B‐cell lymphoma (DLBCL) following glofitamab‐based chemo‐immunotherapy. The patient developed sudden onset altered mental status with extreme hyperammonemia (peak blood ammonia 638.9 µmol/L) despite preserved liver function. Metagenomic next‐generation sequencing (mNGS) of bronchoalveolar lavage fluid identified *Ureaplasma urealyticum*. Furthermore, post‐mortem whole‐exome sequencing (WES) identified a heterozygous variant of SLC25A13 (NM_014251.3:c.2 T > C). As biochemical confirmation of citrin deficiency was not available, the clinical significance of this variant remains uncertain, though it may represent a contributory metabolic susceptibility factor. Despite aggressive ammonia‐lowering strategies, including continuous renal replacement therapy (CRRT) and targeted antibiotics, the patient succumbed to fulminant cerebral edema.

**Conclusion:**

This case highlights the *Ureaplasma urealyticum* infection as a critical precipitant of fatal NHHA following glofitamab therapy, occurring in the background of possible genetic metabolic susceptibility (an unverified heterozygous SLC25A13 variant of uncertain functional significance). These findings underscore the critical need for early blood ammonia monitoring and rapid mNGS screening in immunocompromised patients with unexplained encephalopathy. We propose a structured diagnostic algorithm to expedite the recognition and management of this reversible yet life‐threatening condition.

**Abbreviations**
ALF: acute liver failure
ALT/AST: alanine aminotransferase/aspartate aminotransferase
APTT: activated partial thromboplastin time
BALF: bronchoalveolar lavage fluid
BNP: B‐type natriuretic peptide
BUN: blood urea nitrogen
CRRT: continuous renal replacement therapy
CRS: cytokine release syndrome
CT: computed tomography
DBIL/TBIL: direct bilirubin/total bilirubin
DLBCL: diffuse large B‐cell lymphoma
DNR: do not resuscitate
FDG: fluorodeoxyglucose
FIB: fibrinogen
GCS: Glasgow Coma Scale
GS: glutamine synthetase
Hb: hemoglobin
HTS: hypertonic sodium solutions
ICANS: immune effector cell‐associated neurotoxicity syndrome
ICP: intracranial pressure
ICU: intensive care unit
IL‐6: interleukin‐6
INR: international normalized ratio
IPI: international prognostic index
mNGS: metagenomic next‐generation sequencing
NHHA: non‐hepatic hyperammonemia
PCR: polymerase chain reaction
PET‐CT: positron emission tomography‐computed tomography
PLT: platelet count
PT: prothrombin time
P/F: PaO_2_/FiO_2_ ratio
SLC25A13: solute carrier family 25 member 13 (gene encoding citrin)
TnI: troponin I
WBC: white blood cell count
WES: whole‐exome sequencing
WM: Waldenström macroglobulinemia

## 1 Introduction

Hyperammonemia is conventionally regarded as a hallmark of severe hepatic failure. Non‐hepatic hyperammonemia (NHHA) is uncommon in the intensive care unit (ICU) but progresses rapidly with potent neurotoxicity [1, 2, 3]. Differential diagnosis of NHHA is particularly challenging in patients with hematologic malignancies. Common causes include chemotherapeutic drug toxicity (e.g., asparaginase and 5‐fluorouracil), late‐onset urea cycle disorders in adults, and rare infections by urease‐producing pathogens [4, 5].

NHHA cases have typically been reported in patients who have undergone organ transplantations. Recent studies have identified *Ureaplasma* infection as a key etiology of NHHA [6]. With the widespread application of cellular therapies for autoimmune diseases and malignancies, hyperammonemia in non‐transplant patients caused by systemic infections due to *Ureaplasma urealyticum* (*Ureaplasma* spp.) has been increasingly reported [7]. As this bacterium is not only a commensal flora in the genital tract but is also undetectable in routine blood cultures, it frequently leads to misdiagnosis and missed diagnosis. Through a representative case, this article elucidates the clinical characteristics of this condition and establishes a standardized diagnostic algorithm [7, 8].

## 2 Case Presentation

### 2.1 Admission and Initial Workup

A 58‐year‐old male with a 2‐year history of Waldenström macroglobulinemia (WM) on maintenance zanubrutinib, was admitted on October 8, 2024, presenting with a 20‐day history of persistent high‐grade fever. Admission Chest CT revealed scattered mass‐like shadows in both lungs and multiple enlarged mediastinal lymph nodes.

To investigate the pulmonary lesions, a fiberoptic bronchoscopy was performed on October 9. No endobronchial neoplasms were visualized, and mNGS of the BALF detected only a low copy number of Haemophilus influenzae (20 copies/mL).

### 2.2 Oncological Transformation and Treatment

Given the inconclusive pulmonary workup, a positron emission tomography‐computed tomography (PET‐CT) scan was performed. It revealed generalized lymphadenopathy, multiple space‐occupying lesions in the spleen, and bilateral lung masses with cavitation, all demonstrating significantly increased fluorodeoxyglucose (FDG) metabolism. Notably, diffuse increased FDG uptake was observed throughout the skeletal bone marrow, highly suggestive of extensive malignant infiltration.

To confirm the diagnosis and staging, a supraclavicular lymph node biopsy and bone marrow examination were performed on October 14, 2024. The lymph node biopsy revealed infiltrative B‐cell lymphoma. Comprehensive bone marrow assessment subsequently confirmed the involvement of Non‐Hodgkin B‐cell lymphoma and identified the MYD88 L265P mutation.

Based on these histological, molecular, and imaging findings, the patient was diagnosed with the transformation of WM into Diffuse Large B‐cell Lymphoma (DLBCL) (non‐GCB type, Ann Arbor stage IV B, IPI 4). Following the initial histological confirmation, the rituximab, cyclophosphamide, doxorubicin, vincristine, and prednisone (R‐CHOP) regimen was promptly initiated on October 16, 2024. However, due to refractory disease and suspicion of DLBCL progression, the treatment plan was adjusted in November 2024 to a second‐line regimen consisting of gemcitabine and oxaliplatin (GemOx) combined with glofitamab.

### 2.3 Neurological Crisis and ICU Course

Vital signs on ICU admission were: temperature, 37°C; oxygen supplementation via nasal cannula at 5 L/min with SpO₂ 100%; blood pressure, 135/64 mmHg; respiratory rate, 17 breaths/min; heart rate, 107 bpm. Pupils were 4 mm, equal and round, with an intact light reflex bilaterally.

**FIGURE 1:**
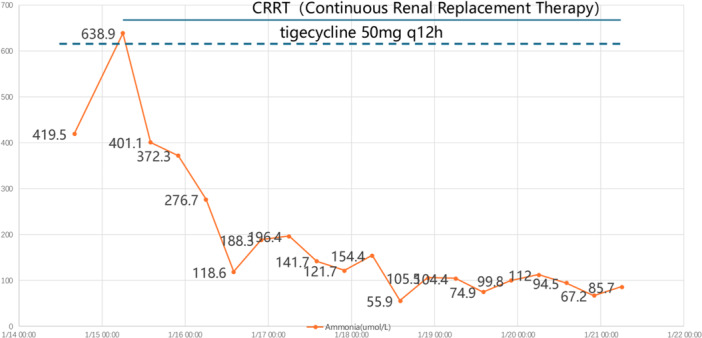
Blood ammonia trend chart. (CRRT January 15, 2025–January 21, 2025, tigecycline 50 mg q12 h January 14, 2025–January 21, 2025).

Laboratory Findings (Table 1): Significant findings included a blood ammonia level of 419.5 µmol/L despite normal liver transaminases and bilirubin levels. Persistent hyperammonemia was observed during the follow‐up (see Figure 1).

**TABLE 1:** Key laboratory findings on the day of ICU admission.

Category	Parameters (units)	Results
Complete blood count	WBC (×10⁹/L); Hb (g/L); PLT (×10⁹/L); N/L (%)	0.9; 69; 10; 76.2/19.3
Inflammation	IL‐6 (pg/mL)	1311
Metabolism	Blood ammonia (µmol/L)	419.5
Cardiac injury	TnI (ng/L); BNP (pg/mL)	20; 679
Electrolytes & renal function	Na/K/Cl (mmol/L); Glucose (mmol/L); BUN (mmol/L); Cr (µmol/L)	153/3.41/116; 10.23; 6.94; 74
Liver function	ALT/AST (U/L); TBIL/DBIL (µmol/L); Albumin (g/L)	27/30; 15.2/3.2; 35.9
Coagulation	PT (s); INR; APTT (s); FIB (g/L)	17.2; 1.43; 38.0; 1.29
Arterial blood gas	pH; PaO₂ (mmHg); PaCO₂ (mmHg); HCO₃⁻ (mmol/L); Lactate (mmol/L); P/F	7.519; 144.0; 24.6; 23.0; 3.5; 351

*Note:* Values are presented as results obtained on the day of ICU admission.

Abbreviations: ALT, alanine aminotransferase; APTT, activated partial thromboplastin time; AST, aspartate aminotransferase; BNP, B‐type natriuretic peptide; BUN, blood urea nitrogen; Cl, chloride; Cr, creatinine; DBIL, direct bilirubin; FIB, fibrinogen; Hb, hemoglobin; HCO₃⁻, bicarbonate; IL‐6, interleukin‐6; INR, international normalized ratio; K, potassium; Na, sodium; N/L, neutrophil/lymphocyte percentage; PaCO₂, arterial partial pressure of carbon dioxide; PaO₂, arterial partial pressure of oxygen; P/F, PaO₂/FiO₂ ratio; PLT, platelet count; PT, prothrombin time; TBIL, total bilirubin; TnI, troponin I; WBC, white blood cell count.

Diagnosis of NHHA: A review of the medication history revealed that no agents were associated with known drug‐induced hyperammonemia. Given the patient's profound immunosuppression status and the literature reports, the team strongly suspected NHHA caused by urease‐producing pathogens (e.g., *Ureaplasma/Mycoplasma hominis*) in immunosuppressed hosts. The antimicrobial regimen was adjusted to tigecycline on January 14, 2025. Fiberoptic bronchoscopy was performed on January 15, 2025. Metagenomic Next‐Generation Sequencing (mNGS) of the bronchoalveolar lavage fluid (BALF) revealed *Ureaplasma urealyticum* on January 16, 2025.

### 2.4 Management and Outcome

Upon ICU admission (January 14, 2025), the patient presented with isolated hyperammonemia without renal failure. Given that severe thrombocytopenia presents a high risk for catheter‐related hemorrhage, and the lack of oliguria, we initially opted for aggressive pharmacological ammonia reduction (l‐ornithine‐l‐aspartate, lactulose). However, follow‐up testing on the early morning of January 15 revealed refractory hyperammonemia. Consequently, we proceeded with continuous renal replacement therapy (CRRT) immediately despite the hemorrhagic risks. Despite these interventions, the patient developed severe bilateral cerebral edema (cranial CT on January 16, 2025) and signs of cerebral herniation (Transcranial Doppler ultrasonography on January 17, 2025). The patient died on January 21, 2025, following the signing of a DNR order by his family. As the patient was critically ill and mechanically supported throughout, tolerability of interventions was assessed clinically by monitoring hemodynamic stability and treatment‐emergent adverse events.

Genetic Susceptibility: The Whole‐exome sequencing (WES) (sample collected on January 17, results returned post‐mortem on February 17) identified a heterozygous variant in the SLC25A13 (located at chr7:95951267, NM_014251.3:c.2 T > C). The functional significance of this variant could not be confirmed in the absence of biochemical validation, though it raises the possibility of a partial predisposition to urea cycle dysfunction.

**FIGURE 2:**
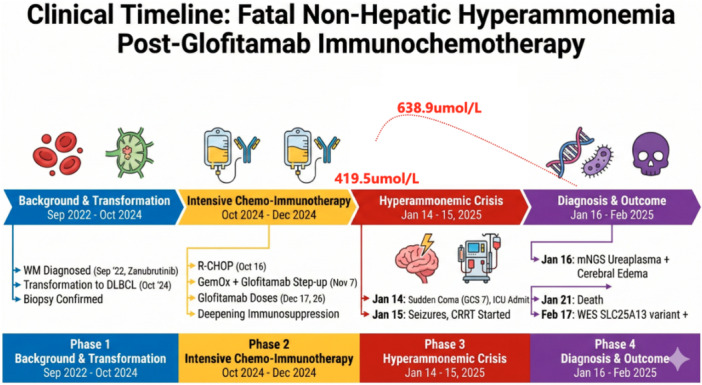
Timeline of the patient.

**FIGURE 3:**
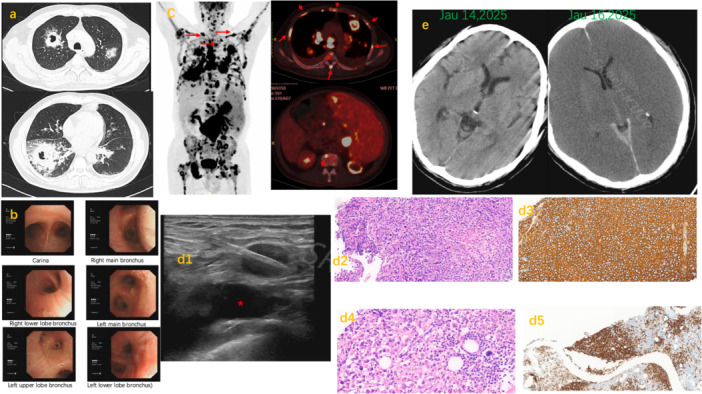
Multimodal imaging, pathological and immunohistochemical findings. (a) October 8, 2024, chest CT showing multiple masses and cavitary lesions. The dominant lesion in the right lower lobe measured approximately 7.1 cm in maximum diameter, with irregular wall thickening (wall thickness 20–30 mm) and no air‐fluid level. (b) October 9, 2024, fiberoptic bronchoscopy showed no endobronchial neoplasms. (c) PET‐CT revealed generalized lymphadenopathy, multiple space‐occupying lesions in the spleen, and bilateral lung masses with cavitation, all demonstrating significantly increased fluorodeoxyglucose (FDG) metabolism. Red arrows indicate the supraclavicular and mediastinal lymph nodes demonstrating significantly increased FDG uptake. (d) Pathological and immunohistochemical findings. (d1) Lymph Node Biopsy: Ultrasound‐guided core needle biopsy of the supraclavicular lymph node (asterisk indicates the subclavian artery). (d2) Lymph Node Histopathology (H&E): Hematoxylin and eosin (H&E) staining of the supraclavicular lymph node biopsy specimen, showing diffuse infiltration of large neoplastic lymphoid cells. (d3) Lymph Node Immunohistochemistry (CD20): Strong and diffuse membranous positivity for CD20 in the lymph node, confirming the B‐cell lineage of the lymphoma. (d4) Bone Marrow Histopathology (H&E): H&E staining of the bone marrow trephine biopsy, illustrating focal infiltration of lymphoma cells. (d5) Bone Marrow Immunohistochemistry (CD20): CD20 staining of the bone marrow specimen, highlighting the involvement of B‐cell lymphoma within the marrow space. (e) Serial cranial CT scans showing the rapid development of diffuse cerebral edema following extreme hyperammonemia. The punctate hyperdense foci represent calcification, as evidenced by their symmetric distribution and absence of perilesional edema.

The patient's clinical course, including the progression of ammonia levels, key therapeutic interventions, and the final outcome, is systematically illustrated in the clinical timeline (Figure 2) and multimodal imaging/pathological findings (Figure 3).

## 3 Discussion

### 3.1 Comprehensive Etiology Hypothesis for NHHA in This Case

Urease‐producing pathogen infection: BALF mNGS definitively detected *Ureaplasma urealyticum*. Combined with the literature‐reported “*Ureaplasma* hyperammonemia syndrome” in transplant recipients and severely immunosuppressed patients, this finding can be considered a significant contributing factor to hyperammonemia [6, 8, 9]. Profound immunosuppression and polymicrobial infections: The patient received long‐term BTK inhibitor and B cell‐targeted antibody therapy, resulting in severely compromised B cell and humoral immunities. Concurrent bacterial/fungal/viral co‐infections create a severe hyperinflammatory state and metabolic burden [9, 10].

The identified heterozygous SLC25A13 variant, if functionally significant, could suggest a partial urea cycle susceptibility background—though this remains unconfirmed without biochemical data. Under conditions of infection, chemotherapy, and nutritional stress, hepatic detoxification capacity is relatively “compressed,” making patients more prone to developing fulminant hyperammonemia within a short time frame [11, 12].

It manifests as a complex form of NHHA, characterized by “profound hyperammonemia without overt hepatic failure, immunosuppression, polymicrobial infections, or suspected metabolic genetic defects.” This case underscores the dilemma in the era of precision medicine: the turnaround time for genetic testing often behind the rapid progression of metabolic crises in ICU settings, highlighting the need for faster, point‐of‐care genomic screening methods.

### 3.2 Immunological Basis of Opportunistic Infection in CD20⁺ B‐Cell Malignancy

The heightened susceptibility to unusual pathogens such as *Ureaplasma urealyticum* in this patient reflects a multilayered immunodeficiency inherent to both the underlying malignancy and its treatment [7, 13]. At the disease level, malignant B‐cell expansion displaces normal B‐cell populations, impairing humoral immunity and antibody‐mediated pathogen clearance [7]. This is frequently compounded by malnutrition and prior infections, all of which further erode innate and adaptive immune defenses. At the treatment level, anti‐CD20 agents such as rituximab and glofitamab deplete peripheral and tissue‐resident B cells through complement‐dependent cytotoxicity, antibody‐dependent cellular cytotoxicity, and phagocytosis [14]. When combined with alkylating agents or purine analogs—as in the R‐CHOP and GemOx regimens received by this patient—additional suppression of T‐cell and myeloid compartments ensues, creating a state of combined humoral and cellular immunodeficiency that substantially broadens the spectrum of opportunistic pathogens capable of causing disseminated, life‐threatening infection [14, 15].

### 3.3 Diagnostic Challenges and Lessons Learned

Prior to initiating a hyperammonemia workup, clinicians should confirm that an elevated ammonia value represents true hyperammonemia rather than a pre‐analytical artifact. Spurious elevations may result from prolonged tourniquet application, delayed sample processing, or failure to transport specimens on ice; repeat testing with proper technique is essential before attributing clinical deterioration to hyperammonemia [16]. Early neurological manifestations (lethargy, incoherent responses, and suspected cortical signs) are easily attributed to cerebral infarction or primary central nervous system involvement, thereby overlooking the need for prompt blood ammonia tests. When liver function indicators are essentially normal with no history of liver disease, the clinical impression that “hyperammonemia must be hepatic encephalopathy” may delay the diagnosis of non‐hepatic hyperammonemia (NHHA) [4, 5]. Conventional cultures and routine antibiotics are ineffective against the cell wall‐deficient *Ureaplasma urealyticum*. Without early molecular methods, such as PCR/mNGS, timely identification of this elusive pathogen remains challenging [7, 8, 17].

### 3.4 Insights Into Treatment Strategies

When blood ammonia levels > 150–200 µmol/L are accompanied by a progressively altered mental status, intensive ammonia‐lowering therapy (CRRT/high‐flux dialysis) should be initiated immediately to address the underlying etiology [4]. It is noteworthy that despite the initiation of CRRT, the rate of serum ammonia clearance was slower than typically observed in hepatic encephalopathy. This likely reflects a “production‐clearance mismatch,” where the continuous, high‐volume production of ammonia by the disseminated *Ureaplasma* load and the potential urea cycle blockade (SLC25A13 variant) outpaced or counteracted the mechanical clearance. Similar refractory patterns have been noted in other severe metabolic crises, suggesting that in such “hyper‐productive” states, earlier or higher‐intensity dialysis doses might be required to overcome the metabolic burden. Given the fulminant cerebral edema that ultimately led to this patient's death, osmotherapy deserves consideration as an adjunctive neuroprotective strategy in severe NHHA. Although direct evidence in the context of non‐hepatic hyperammonemia‐related cerebral edema is lacking, the physiological rationale can be extrapolated from the acute liver failure (ALF) literature. In patients with hepatic encephalopathy, hypertonic sodium solutions (HTS) have been shown to reduce intracranial pressure and decrease brain tissue volume, with one placebo‐controlled study demonstrating that a 3% NaCl bolus targeting serum sodium of 145–155 mEq/L significantly reduced Intracranial Pressure (ICP) compared to placebo [18]. Broader neurocritical care guidelines similarly endorse HTS as a conditional recommendation for ICP management across multiple etiologies of cerebral edema, including hepatic encephalopathy, while acknowledging the very low overall quality of available evidence [19]. In the setting of refractory NHHA with progressive cerebral edema—where ammonia‐lowering capacity is outpaced by ongoing production—prophylactic or early HTS administration may help bridge patients through the metabolic crisis by transiently reducing cerebral edema burden, pending definitive control of the underlying ammonia source. Among patients with hematologic malignancies, transplantation, or profound immunosuppression, coverage for atypical urease‐producing pathogens (such as *Ureaplasma* or *Mycoplasma*) should be initiated prophylactically with agents like doxycycline or fluoroquinolones based on risk factors, with specimens promptly sent for PCR or mNGS testing [8, 9, 17, 20]. For patients with suspected metabolic abnormalities, urea cycle adjuvants (such as arginine and sodium benzoate) may be used in combination; however, individualized decisions should be made based on renal function and specific metabolic assessments [12, 21].

### 3.5 Mechanism of *Ureaplasma*‐Induced Hyperammonemia

*Ureaplasma* spp. is a small bacteria that lack a cell wall, and their metabolism is highly dependent on urea. This bacterium possesses a highly active urease that hydrolyzes urea in body fluids into ammonia (NH3) and carbon dioxide [7, 8]. In immunocompetent hosts, the bacteria are confined to the genitourinary tract. However, in patients with hematologic malignancies, organ transplantation (especially lung transplantation), or long‐term use of humoral immunosuppressants (e.g., rituximab), bacteria can cause disseminated infections, leading to Hyperammonemia Syndrome (HS). In such cases, the ammonia load produced by bacteria in the blood and tissues through the urea cycle far exceeds the metabolic capacity of the liver, resulting in fulminant hyperammonemia [9, 20]. Beyond urease‐mediated ammonia overproduction, an additional mechanism may further amplify the severity of hyperammonemia. Kamel et al. reported that *Ureaplasma* infection is associated with downregulation of glutamine synthetase (GS)—a key enzyme responsible for peripheral ammonia detoxification via glutamine synthesis—in lung transplant recipients who developed hyperammonemia syndrome. This GS downregulation impairs the extrahepatic compensatory pathway that normally buffers excess ammonia, thereby compounding the ammonia accumulation driven by urease activity. This dual mechanism—simultaneous overproduction and impaired peripheral clearance—may partly explain why *Ureaplasma*‐associated hyperammonemia tends to be particularly severe and refractory compared with other non‐hepatic etiologies [22].

### 3.6 Diagnostic Pitfalls and Differential Diagnosis

A hallmark feature in this case was the phenomenon of “dissociation between liver function and blood ammonia levels.” Clinicians often overlook blood ammonia testing because of the absence of a history of liver disease, which can lead to delayed diagnosis. In addition, conventional β‐lactam antibiotics (carbapenems and cephalosporins) that target cell walls are ineffective against *Ureaplasma*, which lacks a cell wall and may mask the condition [7, 23].

### 3.7 Diagnostic and Differential Diagnosis Flowchart Diagnostic Algorithm

**FIGURE 4:**
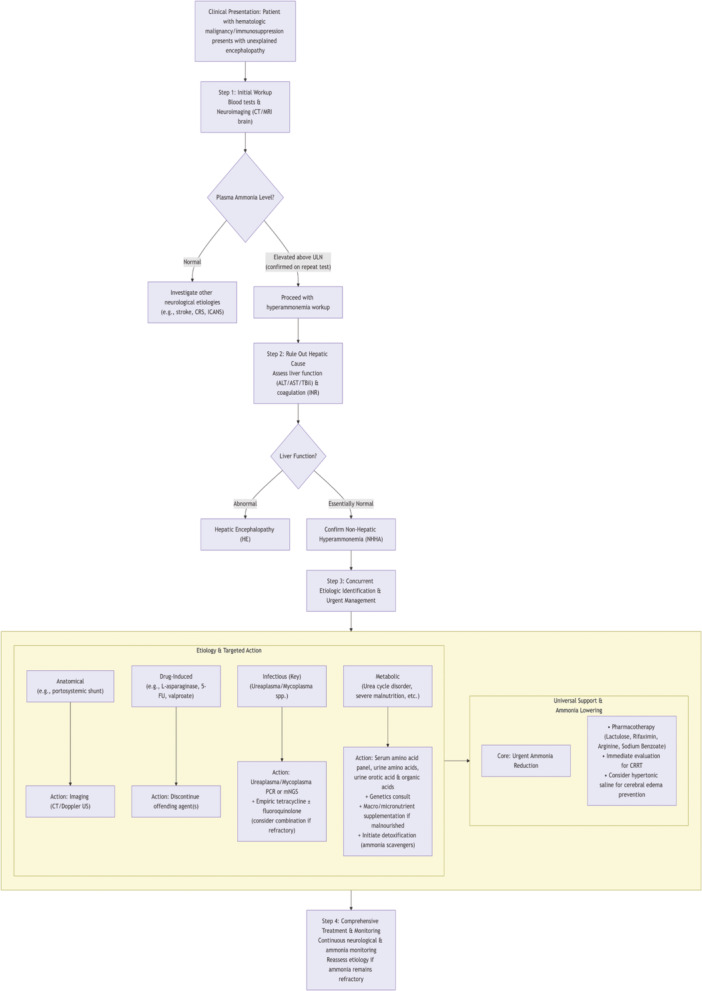
Proposed clinical management pathway to non‐hepatic hyperammonemia (NHHA) in patients presenting with altered mental status post‐ chemo‐immunotherapy.

To enhance ICU physicians' recognition of this condition, we developed a Proposed Clinical Management Pathway for non‐hepatic hyperammonemia (Figure 4).

### 3.8 Limitations

Due to the critically low platelet count, lumbar puncture was contraindicated. Cerebrospinal fluid test results could not be obtained; thus, immune effector cell‐associated neurotoxicity syndrome (ICANS) could not be completely ruled out. Exome sequencing revealed a heterozygous SLC25A13 variant (chr7:95951267, NM_014251.3:c.2 T > C). Heterozygous variants in SLC25A13 are not typically sufficient to cause Citrin deficiency, which generally requires biallelic pathogenic mutations. The potential contribution of this variant to urea cycle dysfunction therefore remains speculative and requires biochemical validation (e.g., serum citrulline, arginine, and ammonia metabolite profiling) to determine its clinical relevance [11, 23]. The WES sample was collected on January 17, 2025, during the crisis, but due to the standard 3‐ to 4‐week turnaround time for WES in our region, results were not available until February 17, 2025—nearly 1 month after the patient's death (January 21, 2025). Consequently, targeted metabolic profiling guided by the genetic findings could not be performed retrospectively during this testing cycle. Therefore, we cannot currently biochemically verify whether the elevation of amino acid metabolic intermediates (such as increased citrulline and arginine concentrations) aligns with the typical metabolic alterations characteristic of Citrin deficiency [11, 12]. As the patient died before the completion of this report, a first‐person patient perspective could not be obtained; the family provided written informed consent for publication on his behalf.

## 4 Conclusion

Hyperammonemia is an independent risk factor for mortality in critically ill patients. Non‐hepatic hyperammonemia (NHHA) may be underrecognized in critically ill patients, and the management of non‐hepatic hyperammonemia occurring post‐ chemo‐immunotherapy in patients with hematologic malignancy involves multidisciplinary collaboration and comprehensive therapeutic strategies. Intensivists must develop a reflex to test blood ammonia in cases of unexplained encephalopathy and, after ruling out common causes, perform PCR testing and initiate combination antibiotic therapy early for suspected *Ureaplasma* infection. Treatment includes aggressive supportive care and measures to reduce ammonia production and enhance its clearance. For *Ureaplasma* infections, empirical therapy with tetracyclines with or without fluoroquinolones may be selected. Blood samples should be collected from patients with an unclear NHHA diagnosis and rapid progression. Exome sequencing can be performed, when necessary, to exclude genetic mutation‐related disorders.

## Author Contributions

**Yinshan Wu:** conceptualization, data curation, formal analysis, investigation, methodology, software, validation, visualization, writing – original draft, writing – review and editing. **Xiuliu Guo:** data curation, investigation, writing – review and editing. **Xiaoling Wang:** data curation, investigation, writing – review and editing. **Feng Guo:** conceptualization, methodology, project administration, resources, supervision, validation, writing – review and editing. All authors read and approved the final article.

## Funding

The authors have nothing to report.

## Ethics Statement

This study is a Case Report and was conducted in accordance with the Declaration of Helsinki. This study (#20262029) was approved by the Medical Ethics Committee of the Sir Run Run Shaw Hospital in January 2026. All figures are original and were created by the authors. The clinical images were obtained during routine patient care and are published with the written informed consent of the patient's family.

## Consent

Written informed consent was obtained from the patient's family to publish this case report and any accompanying images.

## Conflicts of Interest

The authors declare no conflicts of interest.

## Data Availability

The data that support the findings of this study are available from the corresponding author upon reasonable request.
